# Clinical study on the effect of Bi-level positive airway pressure therapy on COPD complicated with Anxiety and Depression

**DOI:** 10.12669/pjms.39.5.7259

**Published:** 2023

**Authors:** Chang-hong Zhang, Jian-hua Liu, Jian-qing Zhao, Zhi-hua Zhang, Xin Gu

**Affiliations:** 1 Chang-hong Zhang, Department of Respiratory and Critical Care Medicine, The First Affiliated Hospital of Hebei North University, Zhangjiakou 075000, Hebei, China; 2 Jian-hua Liu, Department of Respiratory and Critical Care Medicine, The First Affiliated Hospital of Hebei North University, Zhangjiakou 075000, Hebei, China; 3 Jian-qing Zhao, Department of Respiratory and Critical Care Medicine, The First Affiliated Hospital of Hebei North University, Zhangjiakou 075000, Hebei, China; 4 Zhi-hua Zhang, Department of Respiratory and Critical Care Medicine, The First Affiliated Hospital of Hebei North University, Zhangjiakou 075000, Hebei, China; 5 Xin Gu, Department of Neurology, The First Affiliated Hospital of Hebei North University, Zhangjiakou 075000, Hebei, China

**Keywords:** Bi-level positive airway pressure, Chronic obstructive pulmonary disease, Anxiety, Depression

## Abstract

**Objectives:**

To investigate the effect of bi-level positive airway pressure (BIPAP) therapy on chronic obstructive pulmonary disease (COPD) complicated with anxiety and depression.

**Methods:**

This is a retrospective study. One hundred patients with COPD complicated with anxiety and depression who were admitted to the Respiratory Department of The First Affiliated Hospital of Hebei North University from August 2021 to August 2022 were selected and randomly divided into two groups. Patients in the control group were given conventional symptomatic treatment, while those in the observation group were given BIPAP therapy in addition to the treatment in the control group. The two groups were compared and analyzed in terms of respiratory function indicators, the changes in the scores of St. George’s Hospital Respiratory Questionnaire (SGRQ) and COPD assessment test (CAT), blood gas analysis indicators, the levels of serum neurokinin A (NKA), serum interleukin-6 (IL-6), and serum serotonin (5-HT), as well as the changes in the scores of Hamilton Anxiety Scale (HAMA) and Hamilton Depression Scale (HAMD).

**Results:**

After treatment, the levels of lung function indicators in both groups increased, SGRQ and CAT scores decreased, and pH levels remained unchanged. In addition, PaO_2_ levels increased, PCO_2_, 5-HT, NKA and IL-6 levels decreased, and HAMA and HAMD scores decreased. The improvement degree of each indicator in the observation group was superior to that in the control group.

**Conclusion:**

In the clinical treatment of COPD complicated with anxiety and depression, BIPAP boasts effective amelioration of lung function and relief of anxiety and depression symptoms, and its mechanism of action may have a close bearing on ameliorating the levels of 5-HT, NKA, and IL-6 in patients.

## INTRODUCTION

Chronic obstructive pulmonary disease (COPD), as one of the common respiratory diseases, is mainly attributed to lung tissue damage caused by persistent airflow limitation in the lungs, resulting in a series of clinical manifestations such as inability to breathe normally, chest tightness and shortness of breath.^1^ Nevertheless, some patients with COPD still show a situation of chronic disease progression, which eventually leads to progressive weakening of lung function and acute aggravation of symptoms.^2^ Supplemented by long-term hypoxia and carbon dioxide retention, patients may suffer from severe central nervous system dysfunction, which induces a series of mental disorders, among which anxiety and depression are the most common.^3^ In view of this, it is of great clinical significance to take effective measures to relieve anxiety and depression symptoms in patients with COPD.

In recent years, with the use of bi-level positive airway pressure (BIPAP) ventilators, various benefits have been brought such as significant clinical efficacy, greatly reduced endotracheal intubation rate of invasive ventilation in acute exacerbations, hospitalization times of associated pneumonia and acute exacerbations in the stable phase.^4^,^5^ Few studies have been reported at home and abroad on the effect of BIPAP on mental disorders in patients with COPD complicated with anxiety and depression. In this study, the effect of BIPAP therapy on COPD complicated with anxiety and depression was observed, so as to provide more decision-making basis for the selection of clinical protocols for the treatment of COPD complicated with anxiety and depression.

## METHODS

This is a retrospective study. A total of one hundred patients with COPD complicated with anxiety and depression who were admitted to The First Affiliated Hospital of Hebei North University from August 2021 to August 2022 were selected and randomly divided into two groups: the observation group and the control group, with 50 cases in each group. No significant difference was observed in the basic data of patients with COPD complicated with anxiety and depression in the observation group and the control group (*p>*0.05), which were comparable.

### Ethical Approval:

The study was approved by the Institutional Ethics Committee of The First Affiliated Hospital of Hebei North University (No.: K2017150; date: July 15, 2020), and written informed consent was obtained from all participants.

### Inclusion criteria:

- Patients who meet the diagnostic criteria for COPD and anxiety and depression disorders.^6^
- Patients without cognitive impairment and able to communicate with the scale and questionnaires normally.
- Patients who themselves and their families have no objection to the content of this study and signed the informed consent form.

### Exclusion criteria:

- Patients with respiratory system-related diseases other than COPD.
- Patients with severe damage to vital organs of the body such as the heart, brain, liver, and kidney.
- Patients with mental disorders other than anxiety and depression.
- Patients with malignant tumors or patients undergoing malignant tumor surgery.
- Patients already on bronchodilator therapy, malignancy and bronchiectasis.

### Therapeutic methods:

Patients in the control group were given conventional symptomatic treatment (including conventional oxygen therapy, anti-infection, expectorant, antiasthmatic, respiratory function exercise, etc.), commonly used drugs include terbutaline, salbutamol and formoterol, Use in strict accordance with the drug instructions; while those in the observation group were given BIPAP therapy in addition to the treatment in the control group. Mode selection: Spontaneous/timed (S/T). The initial inspiratory pressure was set at 10 cmH2O, gradually increased to 14-20 cmH2O and maintained according to the specific situation of the patient, while the initial expiratory pressure was set at four cmH2O, also gradually increased to six cmH2O and maintained in the same way. The respiratory rate was set at 10-18 times/min, and the patient was ventilated for 5-10 h each time, once a day, two weeks as a course of treatment.

### Observation indicators:

All patients included in this study were tested for various indicators before treatment and after one course of treatment. Duration of follow-up was three months.

### Detection of lung function indicators:

Forced vital capacity (FVC), forced expiratory volume in the first second (FEV1), peak expiratory flow rate (PEF) was detected.

### St. George’s Hospital Respiratory Questionnaire (SGRQ)^7^:

The highest score is 100, and the lower the score, the less the impact of the disease on the patient’s life and the better the patient’s quality of life.

### COPD assessment test (CAT)^8^ scoring scale:

The lower the score, the better the disease control effect.

### Detection of blood gas analysis indicators:

Acid-base (pH), arterial partial pressure of oxygen (PaO_2_), arterial partial pressure of carbon dioxide (PaCO_2_) was detected.

### Detection of serological indicators:

The level of interleukin-6 (IL-6), Serum neurokinin A (NKA) and 5-Hydroxytryptamine (5-HT) were detected.

### Assessment of anxiety and depression:

Hamilton Anxiey Scale (HAMA)^9^ consists of 14 items, each of which is rated on a 5-point scale (0-4 points). The higher the score, the worse the anxiety symptoms. Hamilton Depression Scale (HAMD)^10^ consists of 24 items, of which 14 items are rated on a 5-point scale (0-4 points). The higher the score, the more severe the depressive symptoms.

### Statistical methods:

All count and measurement data in this study were input into SPSS 22.0 software for statistical analysis. The power of test / confidence interval is 95%. For normally distributed measurement data (*X̅*±*S*), differences between groups and within groups were analyzed by independent *t* test and paired *t* test, respectively, and χ^2^ test was used for count data (*n*) to compare differences between groups; *P*<0.05 indicates a statistical significance difference.

## RESULTS

After treatment, the pulmonary function indicators increased, and the indicators in the observation group were higher than those in the control group, with a statistically significant difference (*p<*0.05). Table-I.

**Table-I:** Comparison of pulmonary respiratory function indicators between the two groups (*χ̅*±*S*,L).

Group	Number of cases	FEV1		FVC		PEF	

		Before treatment	After treatment	Before treatment	After treatment	Before treatment	After treatment
Control group	50	1.50±0.23	1.86±0.21*	1.97±0.40	2.39±0.42*	2.79±0.48	3.23±0.62*
Observation group	50	1.48±0.21	2.21±0.31*	1.94±0.49	2.82±0.48*	2.88±0.51	3.76±0.73*
*t*		0.454	6.610	0.335	4.767	0.909	3.913
*p*		0.651	<0.001	0.738	<0.001	0.366	<0.001

***Note:***

* p<0.05 compared with the same group before treatment.

After treatment, SGRQ and CAT scores were decreased, and the scores in the observation group were higher than those in the control group, with a statistically significant difference (*p<*0.05). Table-II. After treatment, there was no significant change in blood pH levels in the two groups, but PaO_2_ levels increased and PCO_2_ levels decreased. The PaO_2_ level in the observation group was higher and the PCO_2_ level was lower in the control group, with a statistically significant difference. Table-III.

**Table-II:** Comparison of SGRQ and CAT scores between the two groups (*χ̅*±*S*, points).

Group	Number of cases	SGRQ		CAT	
	
		Before treatment	After treatment	Before treatment	After treatment
Control group	50	64.80±12.78	45.98±9.35*	27.56±7.79	21.02±2.83*
Observation group	50	65.46±13.86	34.80±7.71*	28.96±6.75	18.52±2.74*
*t*		0.248	6.523	0.960	4.488
*p*		0.805	<0.001	0.339	<0.001

***Note:***

* p<0.05 compared with the same group before treatment.

**Table-III:** Comparison of blood gas analysis indicators between the two groups (*χ̅*±*S*).

Group	Number of cases	PaO_2_ (mmHg)		PCO_2_ (mmHg)		pH	

		Before treatment	After treatment	Before treatment	After treatment	Before treatment	After treatment
Control group	50	56.32±7.80	64.09±7.88*	69.62±7.79	60.30±6.42*	7.31±0.21	7.33±0.13
Observation group	50	57.09±7.52	72.55±10.75*	70.84±8.34	43.98±5.75*	7.30±0.22	7.35±0.11
*t*		0.503	4.488	0.756	13.390	0.232	0.830
*p*		0.616	<0.001	0.452	<0.001	0.817	0.408

***Note:***

* p<0.05 compared with the same group before treatment.

After treatment, the levels of 5-HT, NKA and IL-6 were decreased, and those in the observation group were lower than those in the control group, with statistically significant differences (p<0.05). Table-IV. After treatment, the HAMA and HAMD scores were both decreased, and the scores of the observation group were lower than those of the control group, with statistically significant differences (p<0.05). Table-V.

**Table-IV:** Comparison of serological indicators between the two groups (*χ̅*±*S*).

Group	Number of cases	NKA (pg/mL)		IL-6 (ng/L)		5-HT (ng/mL)	

		Before treatment	After treatment	Before treatment	After treatment	Before treatment	After treatment
Control group	50	366.87±72.32	168.24±35.36*	25.93±5.80	14.56±2.70*	150.49±8.58	110.51±12.87*
Observation group	50	370.26±78.85	103.90±26.69*	25.62±5.36	10.37±1.49*	149.90±9.05	123.40±14.23*
t		0.224	10.269	0.278	9.607	0.335	4.750
p		0.823	<0.001	0.782	<0.001	0.739	<0.001

***Note:***

* p<0.05 compared with the same group before treatment.

**Table-V:** Comparison of anxiety and depression scores between the two groups (*χ̅*±*S*, points).

Group	Number of cases	HAMA		HAMD	

		Before treatment	After treatment	Before treatment	After treatment
Control group	50	23.52±4.99	14.40±2.60*	22.28±5.63	13.50±2.48*
Observation group	50	22.38±4.59	9.92±1.58*	22.68±6.30	10.78±1.67*
*t*		1.189	10.412	0.335	6.433
*p*		0.237	<0.001	0.739	<0.001

***Note:***

* p<0.05 compared with the same group before treatment.

## DISCUSSION

The results of this study were similar to the above reports, suggesting that BIPAP can effectively relieve the anxiety and depression negative emotions of patients with COPD complicated with anxiety and depression, and enable patients to actively participate in the treatment of the disease, which is conducive to improving the clinical efficacy.

The results of this study showed that the levels of 5-HT, NKA and IL-6 in the observation group were lower than those in the control group after treatment. 5-HT, also known as serotonin, exists mostly in the cerebral cortex and synapses. Research^11^ found that serum 5-HT levels in patients with COPD were higher than those in healthy people. Il-6 is the principal inflammatory factor that causes airway inflammatory response and leads to COPD attack by destroying lung structure.^12^ It has been reported^13^ that IL-6 may be a potential pathological factor in major depression. NKA, as a crucial neurogenic inflammatory mediator, is widely distributed in bronchi and lung tissues at all levels.

Anxiety and depression, as a very common complication of COPD, have attracted more and more attention. Research^14^ found that the prevalence of anxiety and depression in COPD patients ranged from 10%-19% and 10%-42%, respectively. BIPAP therapy, as an important means for the treatment of COPD complicated with respiratory failure, is widely used in clinical practice at home and abroad.^15^ According to this study, the improvement of pulmonary respiratory function and blood gas analysis related indicators in the observation group was significantly better than that in the control group after treatment, which was similar to the results of Wang L et al.^16^, suggesting that BIPAP can effectively improve the lung function of patients with COPD complicated with anxiety and depression.

It was also revealed in this study that the reduction of SGRQ and CAT scores in the observation group was better than that in the control group after treatment, which was similar to the results of Sun Jie et al.^17^, suggesting that BIPAP can effectively improve the symptoms of respiratory failure and improve the quality of life of patients with COPD complicated with anxiety and depression. As for the etiology of anxiety and depression in patients with COPD, there is currently no clear standard. It may be due to the mutual influence of a variety of risk factors, resulting in the malignant development of the disease, as well as the brain tissue damage caused by the long-term existence of hypoxemia, CO_2_ retention and acidosis, resulting in the central nervous system organic damage.

All of these will increase the risk of anxiety, depression and other mental disorders.^18^ It was found in this study that the reduction of HAMA and HAMD scores in the observation group after treatment was better than that in the control group, which was similar to the improvement effect on anxiety and depression reported by Zhu M et al.^19^

Relevant studies^20^ have reported that there was a significant correlation between anxiety behaviors and elevated NKA levels. This study, combined with the above reports, all suggest that the mechanism of action of BIPAP on the improvement of COPD symptoms and anxiety and depression mood may have a close bearing on the reduction of 5-HT, NKA and IL-6 levels. However, further animal experiments still need to be carried out to further explore its specific mechanism of action. The conclusion of this study provides reference for the clinical treatment of COPD combined with anxiety and depression.

### Limitations:

It includes a small number of samples were included, and this study was a single-center study, which may have partial data bias. In addition, the specific mechanism of BIPAP has not been clearly analyzed. In response to this, animal experiments are needed in the later stage to further analyze the specific mechanism of BIPAP.

## CONCLUSION

In the clinical treatment of COPD complicated with anxiety and depression, BIPAP boasts effective amelioration of lung function and relief of anxiety and depression symptoms.

### Authors’ Contributions:

**CZ and** **ZZ:** Designed this study, prepared this manuscript, are responsible and accountable for the accuracy and integrity of the work.

**JL and JZ:** Collected and analyzed clinical data.

**XG:** Significantly revised this manuscript.
